# Quality of life of long‐term childhood acute lymphoblastic leukemia survivors: Comparison with healthy controls

**DOI:** 10.1002/pon.6060

**Published:** 2022-11-22

**Authors:** Sofia Chantziara, Jammbe Musoro, Alison C. Rowsell, Charlotte Sleurs, Corneel Coens, Madeline Pe, Stefan Suciu, Michal Kicinski, Pierre Missotten, Els Vandecruys, Anne Uyttebroeck, Marie‐Françoise Dresse, Claire Pluchart, Alina Ferster, Claire Freycon, Jutte Van Der Werff ten Bosch, Pierre Rohrlich, Yves Benoit, Anne‐Sophie Darlington, Caroline Piette, European Organisation for Research and Treatment of Cancer (EORTC) Quality of Life (QLG) and Children's Leukemia Group (CLG)

**Affiliations:** ^1^ School of Health Sciences University of Southampton Southampton UK; ^2^ European Organisation for Research and Treatment of Cancer (EORTC) Brussels Belgium; ^3^ Department of Oncology KU Leuven Leuven Belgium; ^4^ Department of Cognitive Neuropsychology Tilburg University Tilburg The Netherlands; ^5^ Unité de Psychologie de la Sénescence Université de Liège Liège Belgium; ^6^ Department of Pediatric Hematology‐Oncology Ghent University Hospital Ghent Belgium; ^7^ Department of Pediatric Hemato‐Oncology University Hospital Leuven Leuven Belgium; ^8^ Division of Haematology‐Oncology Department of Paediatrics University Hospital Liège and University of Liège Liège Belgium; ^9^ Department of Pediatric Haematology and Oncology CHU Reims Reims France; ^10^ Department of Hemato‐Oncology HUDERF (ULB) Brussels Belgium; ^11^ Department of Pediatric Hematology‐Oncology CHU Grenoble Grenoble France; ^12^ VUB Brussels Belgium; ^13^ Pediatric Oncology CHU Nice Nice France

**Keywords:** adolescent, cancer survivors, child, leukemia, acute lymphoblastic, quality of life, survivorship, young adult

## Abstract

**Objective:**

Improved treatment landscape has led to better outcomes for paediatric acute lymphoblastic leukemia (ALL) survivors. As the number of survivors increase, we need to elucidate the long‐term quality of life (QoL) and domains of complaints in these patients. Furthermore, the main priorities of these patients need to be clarified. We assessed long‐term QoL outcomes of survivors of childhood ALL compared to matched population controls.

**Methods:**

QoL data were collected from survivors recruited in France and Belgium between 2012 and 2017, including the Short Form Health Survey (SF‐12) and the Quality of Life Systemic Inventory (QLSI). The Wilcoxon test was used to compare SF‐12 scale scores between survivors and matched population controls. For the QLSI, comparisons were mainly descriptive.

**Results:**

One hundred and eighty‐six survivors (mean age: 27.6 years; range: 18.1–52.8) at follow‐up completed QoL measures, amongst whom 180 were matched to controls. Overall, survivors had higher QoL on all SF12 scale scores, indicating that they had better functioning compared to controls. Statistically significant differences on the SF12 were observed for Vitality, Social Functioning, Role Limitations due to Emotional Problems and Mental Health scales. QLSI outcomes suggested that survivors were happier than controls with Couple and Social Relations. Controls were unhappiest compared to survivors with Money, Love life, Self‐esteem, Nutrition and Paid Work.

**Conclusions:**

Our findings suggest that survivors of childhood ALL have better QoL outcomes on some domains compared to the general population, specifically around social and emotional functioning, and that they tend to prioritize their relationships more. Interventions for improving QoL outcomes, might build on existing positive experiences with family, friends and partners.

## 1 BACKGROUND

Acute lymphoblastic leukemia (ALL) is the most common childhood malignancy and represents approximately 25% of all childhood cancers^1^ with the diagnosis peaking between 2 and 5 years of age.^2^ Contemporary risk‐directed therapy has led to improved outcomes for paediatric patients with ALL over the last 40 years and survival rates have now reached 90%.^1^ These improvements in survival have increased the need to better understand quality of life (QoL) outcomes among survivors of childhood ALL.

Childhood cancer diagnosis and treatment create significant ongoing physical, social, and emotional challenges for survivors and their families.^3^, ^4^, ^5^, ^6^, ^7^ However, survivors can also describe their illness as having a positive influence on perceptions about self, relationships with others, plans for the future, and life perspectives.^8^, ^9^ The experience of cancer can also push survivors to reassess their life priorities^10^, ^11^ including health and lifestyle, social relationships, and career choices.^10^

Studies investigating QoL outcomes among survivors of childhood ALL report somewhat complex and inconsistent findings, some reporting that ALL survivors had lower QoL than controls^12^, ^13^, ^14^, ^15^, ^16^, ^17^, ^18^ and others describing ALL survivors as having similar or better QoL when compared to healthy controls.^19^, ^20^, ^21^, ^22^, ^23^, ^24^ Their self‐perceptions can change as a result of their diagnosis and treatment^25^ and can learn to adapt to late effects and can re‐examine notions of health and illness.^26^ At the same time, it appears that the effects can be domain‐specific, with survivors reporting more challenges in physical rather than psychological or social aspects of QoL.^14^, ^18^, ^21^

The majority of previous studies have included small samples in terms of size (between *n* = 37 to *n* = 75),^12^, ^13^, ^14^, ^16^, ^17^, ^21^, ^22^ with fewer studies having recruited a larger sample (e.g. *n* > 100).^14^, ^18^, ^20^, ^24^ Previous publications also tend to have used healthy aged‐matched controls^13^, ^14^, ^16^, ^17^ or siblings^18^ as controls. Findings suggest being a sibling to a Childhood Cancer Survivor can impact them,^27^ siblings can report lower HRQoL,^5^, ^7^ including vitality and higher fatigue than healthy controls,^28^ but can also develop psychological resilience, report greater life satisfaction and psychological well‐being through exposure to cancer.^18^ Most studies focused on long‐term outcomes between 5 and 10 years from diagnosis,^4^, ^12^, ^14^, ^15^, ^16^, ^17^, ^19^, ^20^, ^23^, ^24^ or longer‐term outcomes spanning >15 years since treatment.^18^, ^21^, ^22^ Outcomes are likely to be very different for long‐term survivors, who are adults and at a later developmental stage. These survivors will therefore have different life priorities and psychosocial factors will have increasing relevance for them.^28^ It is notable that there is considerable heterogeneity in definitions of survivors and long‐term survivors. Furthermore, the majority of studies evaluated long‐term QoL using health related questionnaires that were originally developed to evaluate health status and do not provide significant breadth to explore QoL issues after the end of treatment. Therefore, we need multi‐domain information to address the full daily life experiences and priorities of survivors. The current study utilized a combination of measures to assess QoL and provides a more nuanced understanding of QoL outcomes among survivors, with a generic QoL questionnaire and a questionnaire that focuses on life priorities. We identified long‐term survivors as adults (not adolescents), with a lengthy follow‐up period compared to other studies,^4^, ^12^, ^14^, ^15^, ^16^, ^17^, ^19^, ^20^, ^23^, ^24^ recognizing that life challenges in adulthood are very different to childhood. The aim of our study was a comprehensive assessment of QoL in a large sample of long‐term survivors of childhood ALL, compared with a matched population of controls.

## 2 METHODS

### 2.1 Design

The current QoL study is part of a larger EORTC study 58LAE^28^ (late adverse effects). This study aims to assess the long‐term outcomes (including QoL, socio‐economic, fertility and medical data) of childhood ALL and lymphoblastic lymphoma survivors who were enrolled as children (<18 years) in the treatment protocols 58,741 (1971–1978), 58,831/2 (1983–1989), and 58,881 (1989–1998) run by the EORTC Children Leukemia Group. Details of the studies have been previously published.^29^ The project was divided in four steps (for details regarding the design, please refer to Piette et al.)^29^: (1) update of the vital status, (2) collection of medical data in the medical records of the patients, (3) collection of socio‐economic data using a patient‐reported questionnaire, and (4) sending of QoL questionnaires for the survivors who answered to the socio‐economic questionnaire. Follow‐up data, including QoL, were collected between 2012 and 2017 from survivors of childhood ALL (≥18 years) recruited from 24 institutions in France and Belgium.

### 2.2 Measures

For the 58LAE study, QoL was assessed via three questionnaires; the Short Form Health Survey (SF‐12),^30^ the Impact of Cancer‐Childhood Survivors^31^ and a questionnaire based on the Quality of Life Systemic Inventory (QLSI).^32^ For the current study, the QoL outcomes based on SF‐12 and QLSI, both completed by ALL survivors and controls, were analyzed.

The SF‐12,^30^ which is the shortened form of the SF‐36, is a generic QoL tool which has 12 items that can be grouped into two dimensions: physical health (Physical Functioning, Role‐Physical, Bodily Pain and General Health) and mental health (Vitality, Social Functioning, Mental Health and Role‐Emotional). A score ranging from 0 (worst possible health) to 100 (optimal health) is obtained for each of them. Studies support the validity and reliability of the SF‐36 when used in long‐term survivors of childhood cancer.^33^ The SF‐12 can replicate accurately the two dimensions of the SF‐36 while also minimizing response burden.^30^

The QoL questionnaire based on the QLSI^32^ contains a combination of 25 items of the adolescent and adult versions of the QLSI. Items represent Life Priorities in different subscales (physical health, cognition, social, couple, leisure, work or school, housekeeping, affectivity and spirituality). Participants are asked to provide a score for each item ranging from 1 (essential) to 5 (not very important) based on the importance they attach to each item. Respondents are also asked to identify five of the 25 items in which they consider themselves the happiest at present and five in which they consider themselves as the unhappiest. We present data comparing the Life Priorities of respondents and controls and the areas of life where they considered themselves to be “the happiest” or “the least happy.” In order to identify domains with highest differences in Life Priorities percentages in the highest end of the scale, *Essential* and *Very Important,* were combined for each item and for each population. QLSI has been shown to have criterion validity and internal consistency among various groups of patients.^34^

### 2.3 Data analysis

The number of patients who completed the SF‐12 and QLSI questionnaires are presented as proportions and absolute numbers. The distribution of the number of completed items within each questionnaire are also presented. Descriptive summaries such as absolute numbers (and percentages), means (unweighted), medians, standard deviations (SD) and ranges were computed. In order to check if the subsample of patients who were assessed for QoL was representative of the overall ALL population in the study, a weighted mean and SD was computed by taking into account proportions of patients cross‐classified by sex, country and age at follow‐up (<18–24 vs. ≥25) in the overall study population (i.e. including ALL survivors who were not assessed for QoL). Comparisons were made between the weighted and unweighted means and SDs for each SF‐12 scale.

Matched control data were collected by *SurveyEngine GmbH,* a company specialized in the conduct of surveys, and maintaining a panel of 110,000 respondents in Belgium and 390,000 in France (https://surveyengine.com/). Control subjects were identified among panel members based on specific pre‐specified criteria (age, province, level of urbanization, and sex) to match the patient profile on our study database. Panel members whose profile matched the requirements were invited to participate in our study using a computer‐ and mobile‐device‐based survey through an anonymous link. Controls were asked to confirm their informed consent electronically before starting to answer the QoL questionnaires. The SF‐12 and QLSI questionnaires were identical to the ones completed by the survivors (except regarding the items influenced by the childhood ALL). Each ALL survivor was matched one‐to‐one to a population control sampled with the same age category (18–19, 20–21, 22–23…36–37, 38–39, 40–44, 45–52), region, level of urbanization (urban area vs. rural area), and sex. A Wilcoxon Rank Sum test was used to test if SF‐12 scale scores differ between matched ALL survivors and population controls since it does not require a normal distribution of the data. To account for multiple testing, *p*‐values below 0.005 were considered to be statistically significant (Bonferroni correction: 0.05/number of tests [=10]).

For the QLSI, comparisons between the Life Priorities of survivors and population controls were mainly descriptive, based on absolute numbers and percentages. QLSI items with the biggest differences between survivors and controls were identified. The analysis was performed in SAS version 9.4^35^ and a complete case analysis strategy was used to handle missing data.

### 2.4 Ethics

At the time of the enrollment in studies 58,741, 58,831/2 and 58,881, informed consent was sought according to local practice of each participating center and in accordance with the Declaration of Helsinki. The EORTC study 58LAE (ClinicalTrials.gov Identifier NCT01298388) was approved by the Ethical Committees of the participating institutions and informed consent was obtained from all participants (patients and controls), in accordance with the applicable national legislation.

## 3 RESULTS

### 3.1 Participants

**FIGURE 1:**
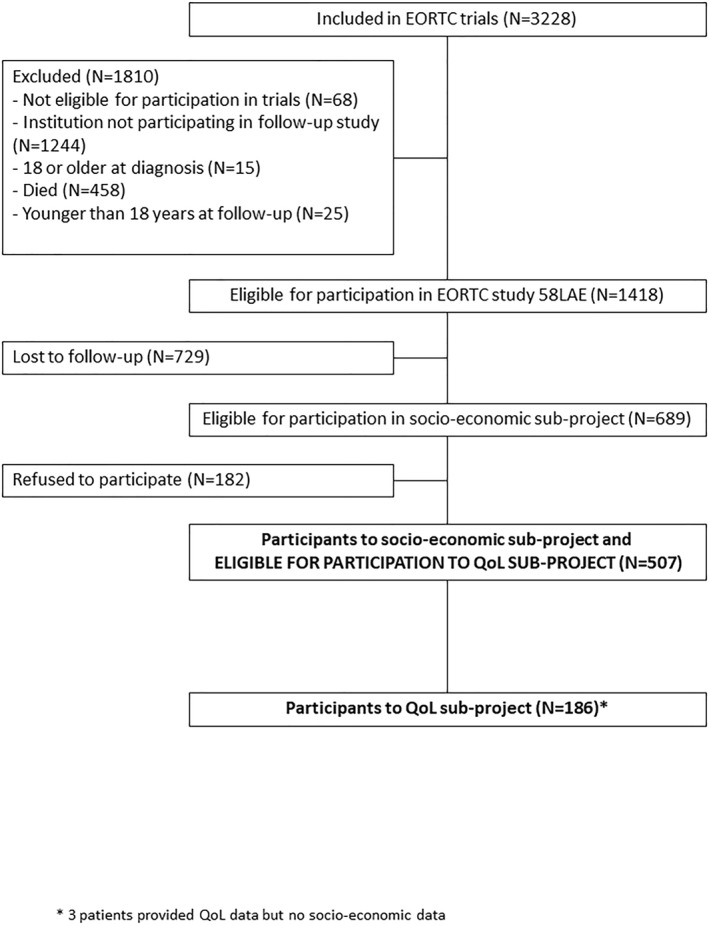
Flow chart of participants. *Three patients provided QoL data but no socio‐economic data. QoL, quality of life

A total of 507 survivors of childhood ALL completed a socio‐economic questionnaire as part of the 58LAE survivorship study, and were eligible for the QoL evaluation (Figure 1). The distribution of disease characteristics was similar between the 507 participants and the patients lost to follow‐up or who refused to participate, except slightly more females among participants (Table S1).

Among the 507 patients eligible for the QoL evaluation, 183 responded (36.1%) and three additional patients provided QoL data but no socio‐economic data, leading to a total of 186 respondents, of which 109 were females (58.6%). The mean age of the survivors at follow‐up was 27.6 years (median: 26.1 and range: 18.1–52.8) and the median time between the diagnosis and the current study was 20.5 years (range: 12.9–41.6). Table S2 also shows a similar distribution of demographic and disease characteristics between survivors who were assessed for QoL and those not assessed for QoL, except an imbalance in the distribution of the participants' country of origin, due to a lower proportion of participating institutions in France compared to Belgium.

Of the 186 ALL survivors who completed at least one of the three QoL questionnaires, 174 filled the SF‐12, with 163 (87.6%) participants completing all items while 11 (6.3%) participants had some items missing. There were 143 survivors who filled the QLSI, with 13 (9.1%) participants completing all items. The proportion of missing items range from 0.7% to 7.7% (Table S3).

### 3.2 QoL of survivors of childhood ALL based on the SF‐12

Tables 1 and 2 present the descriptive summary of the SF‐12 scales scores. The QoL subset was representative of the entire ALL population in our study (Table 2). Unweighted means and SDs for each scale were very close to their weighted estimates (results not shown). For the majority of the scales, difference between weighted versus unweighted means and SDs were within a <1 point range for the SF‐12.

**TABLE 1:** Summary of SF‐12 scale scores for childhood ALL survivors (*N* = 170–173)

	Median	Range	Mean (SD)
Physical functioning	100.0	0.0–100.0	89.75 (23.07)
Role‐physical	87.5	0.0–100.0	81.20 (24.70)
Bodily pain	100.0	0.0–100.0	84.25 (23.83)
General health	85.0	0.0–100.0	72.74 (19.78)
Vitality	75.0	0.0–100.0	60.96 (22.86)
Social functioning	100.0	0.0–100.0	79.36 (25.85)
Role‐emotional	87.5	0.0–100.0	77.95 (24.21)
Mental health	75.0	0.0–100.0	64.82 (20.45)
Physical component summary (range)^a^	57.1 (24.7–67.0)		54.52 (7.87)
Mental component summary (range)^a^	49.5 (18.4–62.5)		47.25 (9.97)

Abbreviations: ALL, acute lymphoblastic leukemia; SD, standard deviation; SF‐12, Short Form Health Survey.

^a^ Apart from these two scales, all other scales had ranges between 0 and 100.

**TABLE 2:** Comparison of SF‐12 scales scores between survivors of childhood ALL and the general population

	ALL survivors (*N* = 164–168)	General population (*N* = 144)	Wilcoxon *p*‐values
Physical functioning			
Median	100.0	100.0	
Range	0.0–100.0	0.0–100.0	
Mean (SD)	89.68 (23.38)	88.54 (20.67)	0.22
Role‐physical			
Median	87.5	75.0	
Range	0.0–100.0	12.5–100.0	
Mean (SD)	81.27 (25.04)	77.34 (23.04)	0.04
Bodily pain			
Median	100.0	75.0	
Range	0.0–100.0	25.0–100.0	
Mean (SD)	84.13 (24.14)	82.47 (20.71)	0.12
General health			
Median	85.0	60.0	
Range	0.0–100.0	25.0–100.0	
Mean (SD)	72.90 (20.00)	69.27 (20.12)	0.08
Vitality			
Median	75.0	50.0	
Range	0.0–100.0	0.0–100.0	
Mean (SD)	61.06 (22.99)	54.51 (19.98)	0.002^a^
Social functioning			
Median	100.0	75.0	
Range	0.0–100.0	0.0–100.0	
Mean (SD)	79.52 (26.01)	70.49 (24.50)	0.0002^a^
Role‐emotional			
Median	87.5	75.0	
Range	0.0–100.0	0.0–100.0	
Mean (SD)	78.28 (24.28)	68.84 (27.46)	0.003^a^
Mental health			
Median	68.8	62.5	
Range	0.0–100.0	25.0–100.0	
Mean (SD)	64.68 (20.40)	60.07 (17.45)	0.01
Physical component summary			
Median	57.1	55.3	
Range	24.7–67.0	33.3–70.8	
Mean (SD)	54.51 (7.97)	54.44 (7.16)	0.2
Mental component summary			
Median	49.5	43.5	
Range	18.4–62.5	20.3–67.2	
Mean (SD)	47.31 (9.98)	43.41 (9.62)	0.0001^a^

*Note*: Higher score indicates better functioning or fewer problems.

Abbreviations: ALL, acute lymphoblastic leukemia; SD, standard deviation; SF‐12, Short Form Health Survey.

^a^ To account for multiple testing, *p*‐values below 0.005 are considered to be statistically significant (i.e. 0.05/number of tests [=10]).

### 3.3 Comparison of QoL between survivors and controls based on the SF‐12

Out of the 186 survivors of childhood ALL who were assessed for QoL, 180 were matched to population controls. Of these matched cases, 168 had completed the SF‐12 questionnaire. ALL survivors had higher scores when compared to matched controls. Statistically significant differences were observed for the *Vitality*, *Social Functioning*, *Role‐Emotional* and *Mental Component Summary* scales (Table 2).

### 3.4 Domains associated with the biggest differences between survivors and controls in the QLSI

Out of the 180 matched ALL survivors and controls who were assessed for QoL, 143 matched cases completed the QLSI questionnaire. Comparisons were made between domains that the patients and controls consider as the most important (i.e. scored as *essential* or *very important*). Furthermore, items for which respondents and controls considered themselves to be “the happiest” or “the least happy” were also compared. Ranking of priorities was performed, to demonstrate the largest differences in chosen priorities between the two groups.

The *Life Priorities* differed between survivors and controls (biggest differences of ≥10% are presented in Table 3, all results are presented in Table S3). A higher proportion of survivors compared to controls prioritized *Intimate relations and Interaction with your friends*. A higher proportion of controls compared to survivors prioritized *Money available to you* (pocket money, student job, allowance, etc.), *Atmosphere/ambience at school/university*; *Your school/university results*, and *Studies.*

**TABLE 3:** Life domains with the biggest differences in priority between survivors and controls

Domains of high priority	ALL survivors		Population controls		Difference
	Number of respondents	%	Number of respondents	%	≥10% (95% confidence limits)
Money available to you (pocket money, student job, allowance, etc.)	38	26.6	90	50	23.4 (13.1, 33.7)
Atmosphere/ambience at school/university	30	21	73	40.6	19.6 (9.9, 29.4)
Studies	37	25.9	81	45	19.1 (8.9, 29.3)
Your school/university results	24	16.8	57	31.6	14.8 (5.7, 24)
Intimate relations (libido; sexuality)	93	65.1	92	51.1	−14 (−24.6, −0.32)
Interaction with your friends	104	72.8	107	59.4	−13.4 (−23.5, −0.31)

*Note*: The number of respondents (%) are based on patients/controls who considered the domains *as essential or very important*.

Abbreviation: ALL, acute lymphoblastic leukemia

We also observed differences in areas where survivors and controls consider themselves as “*the happiest*” or “*the unhappiest*” (biggest differences of ≥10% are presented in Tables 4 and 5, all results are presented in Tables S4 and S5). A higher proportion of survivors compared to controls were “*the happiest*” with *Love Life* and *Interaction with your friends*. A higher proportion of controls compared to survivors were “*the happiest*” with *Sleep*; *Absence of physical pain*; *Relaxing leisure activities; Physical Abilities*. A higher proportion of controls compared to survivors were “*the unhappiest*” with *Love life; Self‐esteem*; *Nutrition*; *Paid Work.* The highest difference (21.23%) between survivors and controls in the domains they consider themselves as “*the unhappiest*” was observed in *Money available to you (pocket money, student job, allowance, etc.).* A higher proportion of controls (*N* = 42; 23.33%) compared to survivors (*N* = 3; 2.10%) were “*the unhappiest*” in this domain.

**TABLE 4:** Life domains with the biggest differences in areas where survivors and controls consider themselves as “the happiest”

Domains in which respondents consider themselves as “the happiest”	ALL survivors		Population controls		Difference
	Number of respondents	%	Number of respondents	%	≥10% (95% confidence limits)
Sleep (ability to sleep well)	19	13.29	57	31.67	−18.38 (−27.2, −9.6)
Absence of physical pain	9	6.29	44	24.44	−18.15 (−25.6, −10.7)
Love life/emotional life/life as a couple (signs of affection, understanding, communication)	77	53.85	71	39.44	14.41 (3.6, 25.3)
Relaxing leisure activities (music, reading, cinema, going out, etc.)	33	23.08	67	37.22	−14.14 (−24, −4.3)
Interaction with your friends	57	39.86	49	27.22	12.64 (2.3, 23)
Physical abilities (ability to walk, climb stairs, etc.)	21	14.69	45	25.00	−10.31 (−18.9, −1.7)

Abbreviation: ALL, acute lymphoblastic leukemia.

**TABLE 5:** Life domains with the biggest differences in areas where survivors and controls consider themselves as “the unhappiest”

Domains in which respondents consider themselves as “the unhappiest”	ALL survivors		Populations controls		Difference
	Number of respondents	%	Number of respondents	%	≥5% (95% confidence limits)
Money available to you (pocket money, student job, allowance, etc.)	3	2.10	42	23.33	−21.23 (−27.9, −14.6)
Love life/emotional life/life as a couple (signs of affection, understanding, communication)	24	16.78	45	25.00	−8.22 (−17, 0.6)
Self‐esteem (overall opinion of yourself)	34	23.78	56	31.11	−7.33 (−17, 2.4)
Paid work	13	9.09	29	16.11	−7.02 (−14.2, 0.1)
Nutrition (type of food etc.)	10	6.99	25	13.89	−6.9 (−13.5, 0.3)

Abbreviation: ALL, acute lymphoblastic leukemia.

## 4 DISCUSSION

The study compared QoL outcomes between ALL long‐term survivors in adulthood, and controls using a combination of measures. The study found that survivors had better outcomes than controls on several QoL domains. Survivors also prioritized and were happier with their relationships, while controls prioritized work, education, and income and were happier with their physical health, sleep and relaxing leisure activities.

More specifically, findings from the SF‐12, which measures physical and mental health, showed significant differences in domains assessing Energy and Vitality; Social Functioning; Role Limitations and Mental Functioning (Mental Component Summary) with survivors having better outcomes than controls. By contrast, a systematic review on outcomes of survivors of childhood ALL showed the majority of studies reported reduced QoL compared to controls.^36^ However, a number of studies have previously shown that ALL survivors can have better or similar QoL compared to controls.^19^, ^20^, ^21^, ^22^, ^23^, ^24^ The current study thus appears to be in line with these findings. Previous studies have also shown that survivors can have better outcomes when compared to controls in more specific domains of psychosocial functioning, while having worse outcomes in physical functioning.^14^, ^18^, ^21^ Findings from the current study are partially in line with previous findings. Survivors in the current study had better psychosocial functioning compared to controls, but they did not appear to have worse outcomes in the perception of their physical functioning. The latter finding could partly be explained by the relatively low proportion of patients treated with cranial radiotherapy or bone marrow/hematopoietic stem cell transplantation in EORTC protocols compared to other treatment protocols.^12^, ^13^, ^14^, ^15^, ^16^, ^17^, ^18^, ^37^, ^38^ Indeed, following the results of the randomized EORTC trial 58,832 (no increase in the incidence of CNS relapse in patients randomized without cranial radiotherapy), the EORTC was the first group to omit cranial radiotherapy as first‐line treatment for all patients with childhood ALL.^39^, ^40^ In the same study, the evaluation of long‐term side effects showed that the omission of cranial radiotherapy was associated with a lower incidence of second neoplasms and a lower rate of late CNS and endocrine adverse events.^40^ Among the three EORTC studies included in the present QoL evaluation, bone marrow/hematopoietic stem cell transplantation was only indicated for “very high‐risk” patients included in EORTC study 58,881, in first complete remission and with an available donor. A recent review found that survivors of childhood hematopoietic stem cell transplantation with a severe chronic health condition, graft versus host disease or pain appear to have poor QOL.^41^ Unfortunately, the small number of patients treated with cranial radiotherapy (*n* = 29; 15.6%) or bone marrow/hematopoietic stem cell transplantation (*n* = 12; 6.5%) among the QoL participants and the existence of confounding factors prevented us from verifying our hypothesis.

The systematic review on survivors of childhood^36^ was able to demonstrate that personal factors, such as the capacity to manage the impact of the disease were related to better outcomes. In particular, having a positive outlook, seeking support and companionship from others were reported as strategies to enhance QoL, which is mirrored in our findings around priorities.

Better QoL outcomes among survivors in the current study could be attributed to survivors re‐evaluating their life priorities. Re‐prioritization processes as seen in the QLSI, such as attributing importance to love‐life and interaction with friends, might have led to improved QoL scores. Previous studies have identified changing life priorities for cancer survivors including around social relationships.^10^, ^11^ Furthermore, participants in the current study were long‐term survivors and therefore, their QoL outcomes may reflect their adaptation to late effects. This replicates previous research which suggests survivors adapt to late effects and reassess their life priorities.^25^, ^26^ In long‐term survivors this adaptation may be more visible.

Differences in QLSI outcomes, although not statistically tested, provided useful insights. Survivors prioritized Relationships more, while controls prioritized Studies, Work and Income. Survivors appeared happier with their Relationships compared to controls while controls were happiest with sleep compared to survivors. Controls were unhappier with the money available to them. Areas where ALL survivors were unhappiest compared to controls did not come into the biggest observed differences; however, memory showed a small difference (Table S5). Previous studies have shown that survivors, as they seek and receive support during their illness, manage to form stronger relationships with family and friends, which they are then able to maintain.^42^ They can also experience benefits in their intimate relationships such as greater appreciation of their partner and increased maturity.^43^ Reporting on life priorities, such as intimate relationships, social aspects of life and studies, as captured in the QLSI, helps to present the survivors' full experience, adding to the more physical and mental and disease specific outcomes measured in traditional QoL measures (e.g. SF12). Studies have also shown negative influences of cancer treatment on cognitive and physical abilities^3^, ^4^, ^5^, ^6^, ^7^, ^14^, ^18^, ^21^, ^44^ which could explain why survivors in the current study did not rate their Physical Health as an area where they considered themselves “happiest” on the QLSI. However, in the current study survivors scored higher than controls in Physical Health on the SF12 (although no statistical difference was reported). One possible reason for this difference, is that the two measures are measuring different aspects of physical health. The QLSI focusses on life priorities asking a general question about assessing how happy respondents are with their physical health. In contrast, the SF12 assesses how physical health limits aspects of life, such as daily activities and work.

### 4.1 Study limitations

The current study had certain limitations. Analysis taking into account the details around the participants socio‐economic status were not carried out. There is some evidence that socio‐economic factors, such as marriage status, employment and low household income are associated with lower QoL.^18^, ^45^ However, the current study lacked information about such socio‐economic factors in the population of healthy controls, hence limiting the choice of matching factors. In addition, the version of the QLSI used in the study was not yet validated and was only used for descriptive purposes, with focus on the items participants considered to be essential or very important and those reported to be the “happiest” or “least happy with.” The age range of participants was quite broad (18–51 year), so it is not possible to specify outcomes for younger or older survivors. Sociodemographic characteristics such as income and ethnicity were not examined in this study. Selection bias may have been present, since survivors sometimes participated decades after diagnosis, which introduces survival bias, with those with most complications of therapy being less likely to have been included. In addition, more survivors were included from Belgium compared to the number included from France, mainly due to the participation of centers in each country.

Study strengths included a focus on long term survivors of childhood ALL in adulthood, recognizing the different life challenges faced by this group as opposed to children or adolescents. The study had a large sample size, and compared adult survivors with a matched population of healthy controls, rather than siblings who might have been impacted by the cancer.

### 4.2 Clinical implications

Practitioners should consider targeting interventions at areas in which survivors appear to have lower QoL or do not see themselves “happiest” in compared to controls. Psycho‐social interventions, that can help survivors identify and re‐evaluate their life priorities and build on existing positive experiences with family, friends and partners might help improve QoL outcomes for this group.

## 5 CONCLUSIONS

Findings from the study indicate that survivors of childhood ALL have better QoL outcomes on some domains when compared to controls especially in domains related to psychosocial functioning. Survivors also prioritize and are happier with their relationships. However, they can also face challenges related to their perceptions of happiness surrounding physical and cognitive functioning and targeted interventions are needed to address their difficulties.

## AUTHOR CONTRIBUTIONS

*Conceptualization*: Charlotte Sleurs, Jammbe Musoro, Alison C. Rowsell, Anne‐Sophie Darlington, Caroline Piette. *Methodology*: Charlotte Sleurs, Alison C. Rowsell, Jammbe Musoro, Anne‐Sophie Darlington, Caroline Piette. *Validation*: Jammbe Musoro. *Formal analysis*: Jammbe Musoro, Corneel Coens, Michal Kicinski, Stefan Suciu. *Resources*: Madeline Pe, Anne‐Sophie Darlington, Caroline Piette. *Writing—original draft preparation*: Charlotte Sleurs, Jammbe Musoro, Alison C. Rowsell, AS, Caroline Piette. *Writing—review and editing*: Charlotte Sleurs, Jammbe Musoro, Alison C. Rowsell, Sofia Chantziara, Corneel Coens, Madeline Pe, Stefan Suciu, Michal Kicinski, Pierre Missotten, Els Vandecruys, Anne Uyttebroeck, Marie‐Françoise Dresse, Claire Pluchart, Alina Ferster, Claire Freycon, Jutte Van Der Werff ten Bosch, Pierre Rohrlich, Yves Benoit, Anne‐Sophie Darlington, Caroline Piette. *Visualization*: Jammbe Musoro. *Supervision*: Anne‐Sophie Darlington, Caroline Piette. *Project administration*: Anne‐Sophie Darlington, Caroline Piette. *Funding acquisition*: Anne‐Sophie Darlington, Caroline Piette. All authors have read and agreed to the published version of the manuscript.

## CONFLICT OF INTEREST

The authors report no conflicts of interest.

## Supporting information

Tables S1–S5

## Data Availability

The data that support the findings of this study are available on request from the corresponding author. The data are not publicly available due to privacy or ethical restrictions.
