# Supplementary material for: Quality of life of long‐term childhood acute lymphoblastic leukemia survivors: Comparison with healthy controls
Source: Psychooncology. 2022 Nov 22;31(12):2159–68. doi: 10.1002/pon.6060 (PMC10099954; doi:10.1002/pon.6060)
Supplement: Supplementary file 1 — Tables S1–S5 [file PON-31-2159-s001.docx]

| **Supplementary Table 1.** Clinical and disease characteristics of patients eligible to the socio-economic evaluation | | | | |
| --- | --- | --- | --- | --- |
|  | **Lost to follow-up (N=729)** | **Refused to participate (N=182)** | **Respondent (N=507)** | **Total (N=1418)** |
|  | **N (%)** | **N (%)** | **N (%)** | **N (%)** |
| **Protocol** |  |  |  |  |
| **N obs** | 729 (100.0) | 182 (100.0) | 507 (100.0) | 1418 (100.0) |
| **58741** | 20 (2.7) | 12 (6.6) | 25 (4.9) | 57 (4.0) |
| **58831** | 199 (27.3) | 44 (24.2) | 109 (21.5) | 352 (24.8) |
| **58881** | 510 (70.0) | 126 (69.2) | 373 (73.6) | 1009 (71.2) |
| **Sex** |  |  |  |  |
| **N obs** | 726 (99.6) | 182 (100.0) | 507 (100.0) | 1415 (99.8) |
| **Male** | 437 (60.2) | 103 (56.6) | 230 (45.4) | 770 (54.4) |
| **Female** | 289 (39.8) | 79 (43.4) | 277 (54.6) | 645 (45.6) |
| **Age at diagnosis, years** |  |  |  |  |
| **N obs** | 726 (99.6) | 182 (100.0) | 507 (100.0) | 1415 (99.8) |
| **<6** | 434 (59.8) | 130 (71.4) | 332 (65.5) | 896 (63.3) |
| **6-9** | 159 (21.9) | 30 (16.5) | 105 (20.7) | 294 (20.8) |
| **10-17** | 133 (18.3) | 22 (12.1) | 70 (13.8) | 225 (15.9) |
| **Country** |  |  |  |  |
| **N obs** | 729 (100.0) | 182 (100.0) | 507 (100.0) | 1418 (100.0) |
| **Belgium** | 259 (35.5) | 106 (58.2) | 236 (46.5) | 601 (42.4) |
| **France** | 470 (64.5) | 76 (41.8) | 271 (53.5) | 817 (57.6) |
| **Disease** |  |  |  |  |
| **N obs** | 723 (99.2) | 182 (100.0) | 506 (99.8) | 1411 (99.5) |
| **ALL** | 645 (89.2) | 182 (100.0) | 498 (98.4) | 1325 (93.9) |
| **NHL** | 78 (10.8) | 0 (0.0) | 8 (1.6) | 86 (6.1) |
| **WBC at diagnosis, x 10⁹/l** |  |  |  |  |
| **N obs** | 724 (99.3) | 181 (99.5) | 507 (100.0) | 1412 (99.6) |
| **<25** | 528 (72.9) | 125 (69.1) | 362 (71.4) | 1015 (71.9) |
| **25 - <50** | 78 (10.8) | 27 (14.9) | 59 (11.6) | 164 (11.6) |
| **≥50** | 118 (16.3) | 29 (16.0) | 86 (17.0) | 233 (16.5) |
| **CNS involvement at diagnosis** |  |  |  |  |
| **N obs** | 708 (97.1) | 179 (98.4) | 505 (99.6) | 1392 (98.2) |
| **CNS-1** | 666 (94.1) | 164 (91.6) | 479 (94.9) | 1309 (94.0) |
| **Not CNS-1** | 42 (5.9) | 15 (8.4) | 26 (5.1) | 83 (6.0) |
| **NCI risk group** |  |  |  |  |
| **N obs** | 724 (99.3) | 181 (99.5) | 507 (100.0) | 1412 (99.6) |
| **Standard Risk** | 496 (68.5) | 133 (73.5) | 358 (70.6) | 987 (69.9) |
| **High Risk** | 228 (31.5) | 48 (26.5) | 149 (29.4) | 425 (30.1) |
| **High treatment intensity per protocol** |  |  |  |  |
| **N obs** | 686 (94.1) | 162 (89.0) | 474 (93.5) | 1322 (93.2) |
| **No** | 583 (85.0) | 129 (79.6) | 407 (85.9) | 1119 (84.6) |
| **Yes** | 103 (15.0) | 33 (20.4) | 67 (14.1) | 203 (15.4) |
| **Relapse within 1 year from diagnosis** |  |  |  |  |
| **N obs** | 729 (100.0) | 182 (100.0) | 507 (100.0) | 1418 (100.0) |
| **No** | 722 (99.0) | 180 (98.9) | 502 (99.0) | 1404 (99.0) |
| **Yes** | 7 (1.0) | 2 (1.1) | 5 (1.0) | 14 (1.0) |

Abbreviations: ALL=acute lymphoblastic leukemia, CNS=central nervous system, HSCT=hematopoietic stem cell transplantation, NCI=national cancer institute, NHL=non-Hodgkin lymphoma, WBC=white blood cell count

| **Supplementary Table 2:** Demographic and clinical characteristics of patients eligible to the QOL evaluation | | | |
| --- | --- | --- | --- |
|  | **QoL Assessed** | | **Total**  **(N=510)**  **N (%)** |
|  | **No (N=324)** | **Yes (N=186)** |  |
|  | **N (%)** | **N (%)** |  |
| **Protocol** |  |  |  |
| **58741** | 8 (2.5) | 17 (9.1) | 25 (4.9) |
| **58831** | 67 (20.7) | 43 (23.1) | 110 (21.6) |
| **58881** | 249 (76.9) | 126 (67.7) | 375 (73.5) |
| **Sex** |  |  |  |
| **Male** | 154 (47.5) | 77 (41.4) | 231 (45.3) |
| **Female** | 170 (52.5) | 109 (58.6) | 279 (54.7) |
| **Age at diagnosis, years** |  |  |  |
| **<6** | 220 (67.9) | 115 (61.8) | 335 (65.7) |
| **>=6** | 104 (32.1) | 71 (38.2) | 175 (34.3) |
| **Range** | 0.3 - 17.9 | 0.2 - 14.7 | 0.2 - 17.9 |
| **Mean (SD)** | 5.43 (3.56) | 5.62 (3.30) | 5.50 (3.47) |
| **Age at follow-up, years** |  |  |  |
| **<18-24** | 158 (48.8) | 85 (45.7) | 243 (47.6) |
| **>=25** | 166 (51.2) | 101 (54.3) | 267 (52.4) |
| **Range** | 18.1 - 51.1 | 18.1 - 52.8 | 18.1 - 52.8 |
| **Mean (SD)** | 25.98 (5.60) | 27.61 (7.09) | 26.57 (6.23) |
| **Country** |  |  |  |
| **Belgium** | 89 (27.5) | 148 (79.6) | 237 (46.5) |
| **France** | 235 (72.5) | 38 (20.4) | 273 (53.5) |
| **WBC at diagnosis** |  |  |  |
| **<25** | 239 (73.8) | 125 (67.2) | 364 (71.4) |
| **>=25** | 85 (26.2) | 61 (32.8) | 146 (28.6) |
| **NCI risk group** |  |  |  |
| **Standard Risk** | 230 (71.0) | 131 (70.4) | 361 (70.8) |
| **High Risk** | 94 (29.0) | 55 (29.6) | 149 (29.2) |
| **HSCT** |  |  |  |
| **No** | 298 (92.0) | 174 (93.5) | 472 (92.5) |
| **Yes** | 26 (8.0) | 12 (6.5) | 38 (7.5) |
| **Relapse** |  |  |  |
| **No** | 282 (87.0) | 161 (86.6) | 443 (86.9) |
| **Yes** | 42 (13.0) | 25 (13.4) | 67 (13.1) |
| **Relapse or Second cancer** |  |  |  |
| **No** | 274 (84.6) | 151 (81.2) | 425 (83.3) |
| **Yes** | 50 (15.4) | 35 (18.8) | 85 (16.7) |
| **Cranial radiotherapy** |  |  |  |
| **No** | 292 (90.1) | 157 (84.4) | 449 (88.0) |
| **Yes** | 32 (9.9) | 29 (15.6) | 61 (12.0) |
| **Being married or living with a partner** |  |  |  |
| **No** | 200 (61.7) | 112 (60.2) | 312 (61.2) |
| **Yes** | 122 (37.7) | 69 (37.1) | 191 (37.5) |
| **Missing** | 2 (0.6) | 5 (2.7) | 7 (1.4) |
| **University degree** |  |  |  |
| **No** | 155 (47.8) | 85 (45.7) | 240 (47.1) |
| **Yes** | 164 (50.6) | 95 (51.1) | 259 (50.8) |
| **Missing** | 5 (1.5) | 6 (3.2) | 11 (2.2) |
| **Currently working** |  |  |  |
| **No** | 124 (38.3) | 57 (30.6) | 181 (35.5) |
| **Yes** | 197 (60.8) | 125 (67.2) | 322 (63.1) |
| **Missing** | 3 (0.9) | 4 (2.2) | 7 (1.4) |

Abbreviations: SD = standard deviation; HSCT= hematopoietic stem cell transplantation; WBC: White Blood Cell Count; NCI: National Cancer Institute

| **Supplementary Table 3:** Comparison of QLSI items between matched survivors and controls | | | |
| --- | --- | --- | --- |
|  | **Groups** | | **Total (N=323)** |
|  | **Population controls (N=180)** | **Childhood ALL survivors (N=143)** |  |
|  | **N (%)** | **N (%)** | **N (%)** |
| **Sleep (ability to sleep well)** |  |  |  |
| **Essential** | 94 (52.2) | 48 (33.6) | 142 (44.0) |
| **Very important** | 57 (31.7) | 59 (41.3) | 116 (35.9) |
| **Important** | 26 (14.4) | 30 (21.0) | 56 (17.3) |
| **Moderately important** | 3 (1.7) | 2 (1.4) | 5 (1.5) |
| **Not very important or not important** | 0 (0.0) | 3 (2.1) | 3 (0.9) |
| **Missing** | 0 (0.0) | 1 (0.7) | 1 (0.3) |
| **Vitality (Having energy to do things)** |  |  |  |
| **Essential** | 59 (32.8) | 51 (35.7) | 110 (34.1) |
| **Very important** | 84 (46.7) | 61 (42.7) | 145 (44.9) |
| **Important** | 33 (18.3) | 27 (18.9) | 60 (18.6) |
| **Moderately important** | 3 (1.7) | 1 (0.7) | 4 (1.2) |
| **Not very important or not important** | 0 (0.0) | 1 (0.7) | 1 (0.3) |
| **Missing** | 1 (0.6) | 2 (1.4) | 3 (0.9) |
| **Physical abilities (ability to walk, climb stairs, etc.)** |  |  |  |
| **Essential** | 73 (40.6) | 52 (36.4) | 125 (38.7) |
| **Very important** | 73 (40.6) | 60 (42.0) | 133 (41.2) |
| **Important** | 27 (15.0) | 23 (16.1) | 50 (15.5) |
| **Moderately important** | 5 (2.8) | 5 (3.5) | 10 (3.1) |
| **Not very important or not important** | 1 (0.6) | 1 (0.7) | 2 (0.6) |
| **Missing** | 1 (0.6) | 2 (1.4) | 3 (0.9) |
| **Nutrition (type of food, etc.)** |  |  |  |
| **Essential** | 34 (18.9) | 28 (19.6) | 62 (19.2) |
| **Very important** | 75 (41.7) | 59 (41.3) | 134 (41.5) |
| **Important** | 47 (26.1) | 47 (32.9) | 94 (29.1) |
| **Moderately important** | 17 (9.4) | 7 (4.9) | 24 (7.4) |
| **Not very important or not important** | 5 (2.8) | 0 (0.0) | 5 (1.5) |
| **Missing** | 2 (1.1) | 2 (1.4) | 4 (1.2) |
| **Absence of physical pain** |  |  |  |
| **Essential** | 31 (17.2) | 29 (20.3) | 60 (18.6) |
| **Very important** | 83 (46.1) | 70 (49.0) | 153 (47.4) |
| **Important** | 52 (28.9) | 34 (23.8) | 86 (26.6) |
| **Moderately important** | 9 (5.0) | 5 (3.5) | 14 (4.3) |
| **Not very important or not important** | 3 (1.7) | 3 (2.1) | 6 (1.9) |
| **Not applicable** | 1 (0.6) | 0 (0.0) | 1 (0.3) |
| **Missing** | 1 (0.6) | 2 (1.4) | 3 (0.9) |
| **Overall physical health** |  |  |  |
| **Essential** | 45 (25.0) | 44 (30.8) | 89 (27.6) |
| **Very important** | 87 (48.3) | 66 (46.2) | 153 (47.4) |
| **Important** | 34 (18.9) | 29 (20.3) | 63 (19.5) |
| **Moderately important** | 10 (5.6) | 1 (0.7) | 11 (3.4) |
| **Not very important or not important** | 1 (0.6) | 1 (0.7) | 2 (0.6) |
| **Missing** | 3 (1.7) | 2 (1.4) | 5 (1.5) |
| **Relaxing leisure activities (music, reading, cinema, going out, etc.)** |  |  |  |
| **Essential** | 30 (16.7) | 31 (21.7) | 61 (18.9) |
| **Very important** | 64 (35.6) | 55 (38.5) | 119 (36.8) |
| **Important** | 67 (37.2) | 43 (30.1) | 110 (34.1) |
| **Moderately important** | 14 (7.8) | 12 (8.4) | 26 (8.0) |
| **Not very important or not important** | 5 (2.8) | 0 (0.0) | 5 (1.5) |
| **Missing** | 0 (0.0) | 2 (1.4) | 2 (0.6) |
| **Active leisure activities requiring energy (sport, gardening, etc.)** |  |  |  |
| **Essential** | 16 (8.9) | 20 (14.0) | 36 (11.1) |
| **Very important** | 44 (24.4) | 40 (28.0) | 84 (26.0) |
| **Important** | 70 (38.9) | 57 (39.9) | 127 (39.3) |
| **Moderately important** | 36 (20.0) | 22 (15.4) | 58 (18.0) |
| **Not very important or not important** | 12 (6.7) | 2 (1.4) | 14 (4.3) |
| **Not applicable** | 1 (0.6) | 0 (0.0) | 1 (0.3) |
| **Missing** | 1 (0.6) | 2 (1.4) | 3 (0.9) |
| **Interaction with your children** |  |  |  |
| **Essential** | 35 (19.4) | 39 (27.3) | 74 (22.9) |
| **Very important** | 32 (17.8) | 24 (16.8) | 56 (17.3) |
| **Important** | 13 (7.2) | 15 (10.5) | 28 (8.7) |
| **Moderately important** | 12 (6.7) | 4 (2.8) | 16 (5.0) |
| **Not very important or not important** | 6 (3.3) | 3 (2.1) | 9 (2.8) |
| **Not applicable** | 79 (43.9) | 56 (39.2) | 135 (41.8) |
| **Missing** | 3 (1.7) | 2 (1.4) | 5 (1.5) |
| **Interaction with your family (parents, siblings, etc.)** |  |  |  |
| **Essential** | 50 (27.8) | 48 (33.6) | 98 (30.3) |
| **Very important** | 73 (40.6) | 52 (36.4) | 125 (38.7) |
| **Important** | 32 (17.8) | 27 (18.9) | 59 (18.3) |
| **Moderately important** | 20 (11.1) | 11 (7.7) | 31 (9.6) |
| **Not very important or not important** | 4 (2.2) | 3 (2.1) | 7 (2.2) |
| **Missing** | 1 (0.6) | 2 (1.4) | 3 (0.9) |
| **Interaction with your friends** |  |  |  |
| **Essential** | 31 (17.2) | 33 (23.1) | 64 (19.8) |
| **Very important** | 76 (42.2) | 71 (49.7) | 147 (45.5) |
| **Important** | 52 (28.9) | 32 (22.4) | 84 (26.0) |
| **Moderately important** | 19 (10.6) | 3 (2.1) | 22 (6.8) |
| **Not very important or not important** | 1 (0.6) | 2 (1.4) | 3 (0.9) |
| **Missing** | 1 (0.6) | 2 (1.4) | 3 (0.9) |
| **Paid work** |  |  |  |
| **Essential** | 58 (32.2) | 33 (23.1) | 91 (28.2) |
| **Very important** | 58 (32.2) | 61 (42.7) | 119 (36.8) |
| **Important** | 34 (18.9) | 17 (11.9) | 51 (15.8) |
| **Moderately important** | 11 (6.1) | 5 (3.5) | 16 (5.0) |
| **Not very important or not important** | 2 (1.1) | 1 (0.7) | 3 (0.9) |
| **Not applicable** | 13 (7.2) | 16 (11.2) | 29 (9.0) |
| **Missing** | 4 (2.2) | 10 (7.0) | 14 (4.3) |
| **Efficiency at work** |  |  |  |
| **Essential** | 34 (18.9) | 23 (16.1) | 57 (17.6) |
| **Very important** | 59 (32.8) | 60 (42.0) | 119 (36.8) |
| **Important** | 58 (32.2) | 31 (21.7) | 89 (27.6) |
| **Moderately important** | 13 (7.2) | 2 (1.4) | 15 (4.6) |
| **Not very important or not important** | 2 (1.1) | 1 (0.7) | 3 (0.9) |
| **Not applicable** | 12 (6.7) | 15 (10.5) | 27 (8.4) |
| **Missing** | 2 (1.1) | 11 (7.7) | 13 (4.0) |
| **Atmosphere/ambience at work** |  |  |  |
| **Essential** | 27 (15.0) | 27 (18.9) | 54 (16.7) |
| **Very important** | 83 (46.1) | 63 (44.1) | 146 (45.2) |
| **Important** | 40 (22.2) | 22 (15.4) | 62 (19.2) |
| **Moderately important** | 8 (4.4) | 4 (2.8) | 12 (3.7) |
| **Not very important or not important** | 1 (0.6) | 1 (0.7) | 2 (0.6) |
| **Not applicable** | 18 (10.0) | 16 (11.2) | 34 (10.5) |
| **Missing** | 3 (1.7) | 10 (7.0) | 13 (4.0) |
| **Financial Matters (incomes, allowance, etc.)** |  |  |  |
| **Essential** | 44 (24.4) | 32 (22.4) | 76 (23.5) |
| **Very important** | 62 (34.4) | 52 (36.4) | 114 (35.3) |
| **Important** | 44 (24.4) | 29 (20.3) | 73 (22.6) |
| **Moderately important** | 14 (7.8) | 7 (4.9) | 21 (6.5) |
| **Not applicable** | 13 (7.2) | 15 (10.5) | 28 (8.7) |
| **Missing** | 3 (1.7) | 8 (5.6) | 11 (3.4) |
| **Where you live (home, neighborhood, surroundings, etc.)** |  |  |  |
| **Essential** | 36 (20.0) | 28 (19.6) | 64 (19.8) |
| **Very important** | 70 (38.9) | 52 (36.4) | 122 (37.8) |
| **Important** | 55 (30.6) | 52 (36.4) | 107 (33.1) |
| **Moderately important** | 11 (6.1) | 5 (3.5) | 16 (5.0) |
| **Not very important or not important** | 3 (1.7) | 3 (2.1) | 6 (1.9) |
| **Missing** | 5 (2.8) | 3 (2.1) | 8 (2.5) |
| **Upkeep of the house/flat** |  |  |  |
| **Essential** | 22 (12.2) | 15 (10.5) | 37 (11.5) |
| **Very important** | 51 (28.3) | 54 (37.8) | 105 (32.5) |
| **Important** | 71 (39.4) | 57 (39.9) | 128 (39.6) |
| **Moderately important** | 27 (15.0) | 10 (7.0) | 37 (11.5) |
| **Not very important or not important** | 5 (2.8) | 4 (2.8) | 9 (2.8) |
| **Not applicable** | 1 (0.6) | 0 (0.0) | 1 (0.3) |
| **Missing** | 3 (1.7) | 3 (2.1) | 6 (1.9) |
| **Memory (ability to remember things to do, some words, past and future events, etc.)** |  |  |  |
| **Essential** | 58 (32.2) | 30 (21.0) | 88 (27.2) |
| **Very important** | 74 (41.1) | 71 (49.7) | 145 (44.9) |
| **Important** | 38 (21.1) | 35 (24.5) | 73 (22.6) |
| **Moderately important** | 7 (3.9) | 5 (3.5) | 12 (3.7) |
| **Not very important or not important** | 2 (1.1) | 0 (0.0) | 2 (0.6) |
| **Missing** | 1 (0.6) | 2 (1.4) | 3 (0.9) |
| **Concentration and attention** |  |  |  |
| **Essential** | 31 (17.2) | 18 (12.6) | 49 (15.2) |
| **Very important** | 87 (48.3) | 71 (49.7) | 158 (48.9) |
| **Important** | 52 (28.9) | 47 (32.9) | 99 (30.7) |
| **Moderately important** | 8 (4.4) | 4 (2.8) | 12 (3.7) |
| **Not very important or not important** | 1 (0.6) | 0 (0.0) | 1 (0.3) |
| **Missing** | 1 (0.6) | 3 (2.1) | 4 (1.2) |
| **Self-esteem (overall opinion of yourself)** |  |  |  |
| **Essential** | 47 (26.1) | 28 (19.6) | 75 (23.2) |
| **Very important** | 83 (46.1) | 65 (45.5) | 148 (45.8) |
| **Important** | 39 (21.7) | 37 (25.9) | 76 (23.5) |
| **Moderately important** | 9 (5.0) | 9 (6.3) | 18 (5.6) |
| **Not very important or not important** | 1 (0.6) | 1 (0.7) | 2 (0.6) |
| **Missing** | 1 (0.6) | 3 (2.1) | 4 (1.2) |
| **Morale** |  |  |  |
| **Essential** | 48 (26.7) | 28 (19.6) | 76 (23.5) |
| **Very important** | 69 (38.3) | 66 (46.2) | 135 (41.8) |
| **Important** | 50 (27.8) | 38 (26.6) | 88 (27.2) |
| **Moderately important** | 11 (6.1) | 9 (6.3) | 20 (6.2) |
| **Not very important or not important** | 1 (0.6) | 0 (0.0) | 1 (0.3) |
| **Missing** | 1 (0.6) | 2 (1.4) | 3 (0.9) |
| **Peace of mind (not being worried, anxious or preoccupied or upset)** |  |  |  |
| **Essential** | 38 (21.1) | 25 (17.5) | 63 (19.5) |
| **Very important** | 94 (52.2) | 69 (48.3) | 163 (50.5) |
| **Important** | 36 (20.0) | 35 (24.5) | 71 (22.0) |
| **Moderately important** | 9 (5.0) | 9 (6.3) | 18 (5.6) |
| **Not very important or not important** | 1 (0.6) | 3 (2.1) | 4 (1.2) |
| **Missing** | 2 (1.1) | 2 (1.4) | 4 (1.2) |
| **Love life/emotional life/life as a couple(signs of affection, understanding, communication)** |  |  |  |
| **Essential** | 47 (26.1) | 52 (36.4) | 99 (30.7) |
| **Very important** | 68 (37.8) | 50 (35.0) | 118 (36.5) |
| **Important** | 46 (25.6) | 28 (19.6) | 74 (22.9) |
| **Moderately important** | 11 (6.1) | 5 (3.5) | 16 (5.0) |
| **Not very important or not important** | 2 (1.1) | 3 (2.1) | 5 (1.5) |
| **Not applicable** | 5 (2.8) | 0 (0.0) | 5 (1.5) |
| **Missing** | 1 (0.6) | 5 (3.5) | 6 (1.9) |
| **Intimate relations (libido, sexuality)** |  |  |  |
| **Essential** | 31 (17.2) | 28 (19.6) | 59 (18.3) |
| **Very important** | 61 (33.9) | 65 (45.5) | 126 (39.0) |
| **Important** | 58 (32.2) | 39 (27.3) | 97 (30.0) |
| **Moderately important** | 21 (11.7) | 6 (4.2) | 27 (8.4) |
| **Not very important or not important** | 3 (1.7) | 3 (2.1) | 6 (1.9) |
| **Not applicable** | 5 (2.8) | 0 (0.0) | 5 (1.5) |
| **Missing** | 1 (0.6) | 2 (1.4) | 3 (0.9) |
| **Spiritual, philosophical or religious life** |  |  |  |
| **Essential** | 13 (7.2) | 6 (4.2) | 19 (5.9) |
| **Very important** | 15 (8.3) | 19 (13.3) | 34 (10.5) |
| **Important** | 27 (15.0) | 30 (21.0) | 57 (17.6) |
| **Moderately important** | 46 (25.6) | 46 (32.2) | 92 (28.5) |
| **Not very important or not important** | 66 (36.7) | 40 (28.0) | 106 (32.8) |
| **Not applicable** | 12 (6.7) | 0 (0.0) | 12 (3.7) |
| **Missing** | 1 (0.6) | 2 (1.4) | 3 (0.9) |
| **Studies** |  |  |  |
| **Essential** | 34 (18.9) | 15 (10.5) | 49 (15.2) |
| **Very important** | 47 (26.1) | 22 (15.4) | 69 (21.4) |
| **Important** | 57 (31.7) | 17 (11.9) | 74 (22.9) |
| **Moderately important** | 11 (6.1) | 2 (1.4) | 13 (4.0) |
| **Not very important or not important** | 3 (1.7) | 1 (0.7) | 4 (1.2) |
| **Not applicable** | 23 (12.8) | 76 (53.1) | 99 (30.7) |
| **Missing** | 5 (2.8) | 10 (7.0) | 15 (4.6) |
| **Your school/university results** |  |  |  |
| **Essential** | 17 (9.4) | 10 (7.0) | 27 (8.4) |
| **Very important** | 40 (22.2) | 14 (9.8) | 54 (16.7) |
| **Important** | 67 (37.2) | 29 (20.3) | 96 (29.7) |
| **Moderately important** | 19 (10.6) | 1 (0.7) | 20 (6.2) |
| **Not very important or not important** | 3 (1.7) | 0 (0.0) | 3 (0.9) |
| **Not applicable** | 27 (15.0) | 78 (54.5) | 105 (32.5) |
| **Missing** | 7 (3.9) | 11 (7.7) | 18 (5.6) |
| **Atmosphere/ambience at school/university** |  |  |  |
| **Essential** | 19 (10.6) | 7 (4.9) | 26 (8.0) |
| **Very important** | 54 (30.0) | 23 (16.1) | 77 (23.8) |
| **Important** | 43 (23.9) | 17 (11.9) | 60 (18.6) |
| **Moderately important** | 17 (9.4) | 4 (2.8) | 21 (6.5) |
| **Not very important or not important** | 2 (1.1) | 1 (0.7) | 3 (0.9) |
| **Not applicable** | 37 (20.6) | 81 (56.6) | 118 (36.5) |
| **Missing** | 8 (4.4) | 10 (7.0) | 18 (5.6) |
| **Money available to you (pocket money, student job, allowance, etc.)** |  |  |  |
| **Essential** | 30 (16.7) | 17 (11.9) | 47 (14.6) |
| **Very important** | 60 (33.3) | 21 (14.7) | 81 (25.1) |
| **Important** | 52 (28.9) | 15 (10.5) | 67 (20.7) |
| **Moderately important** | 14 (7.8) | 3 (2.1) | 17 (5.3) |
| **Not very important or not important** | 2 (1.1) | 0 (0.0) | 2 (0.6) |
| **Not applicable** | 15 (8.3) | 79 (55.2) | 94 (29.1) |
| **Missing** | 7 (3.9) | 8 (5.6) | 15 (4.6) |
|  |  |  |  |
|  |  |  |  |

| **Supplementary Table 4:** Life domains in which respondents consider themselves as "the happiest" | | | | |
| --- | --- | --- | --- | --- |
|  | **ALL survivors** | | **Populations controls** | |
| **Domains ("the happiest")** | Number of respondent | % | Number of respondent | % |
| Love life/emotional life/life as a couple(signs of affection, understanding, communication) | 77 | 53.85 | 71 | 39.44 |
| Interaction with your family (parents, siblings, etc.) | 68 | 47.55 | 72 | 40.00 |
| Interaction with your friends | 57 | 39.86 | 49 | 27.22 |
| Overall physical health | 48 | 33.57 | 50 | 27.78 |
| Paid work | 40 | 27.97 | 44 | 24.44 |
| Interaction with your children | 36 | 25.17 | 28 | 15.56 |
| Where you live (home, neighbourhood, surroundings, etc.) | 36 | 25.17 | 43 | 23.89 |
| Relaxing leisure activities (music, reading, cinema, going out, etc.) | 33 | 23.08 | 67 | 37.22 |
| Atmosphere/ambience at work | 23 | 16.08 | 25 | 13.89 |
| Vitality (Having energy to do things) | 23 | 16.08 | 29 | 16.11 |
| Intimate relations (libido, sexuality) | 22 | 15.38 | 23 | 12.78 |
| Physical abilities (ability to walk, climb stairs, etc.) | 21 | 14.69 | 45 | 25.00 |
| Sleep (ability to sleep well) | 19 | 13.29 | 57 | 31.67 |
| Financial Matters (incomes, allowance, etc.) | 18 | 12.59 | 22 | 12.22 |
| Active leisure activities requiring energy (sport, gardening, etc.) | 16 | 11.19 | 26 | 14.44 |
| Self-esteem (overall opinion of yourself) | 16 | 11.19 | 17 | 9.44 |
| Peace of mind (not being worried, anxious or preoccupied or upset) | 15 | 10.49 | 15 | 8.33 |
| Efficiency at work | 14 | 9.79 | 10 | 5.56 |
| Moral | 14 | 9.79 | 10 | 5.56 |
| Nutrition (type of food, etc.) | 12 | 8.39 | 33 | 18.33 |
| Absence of physical pain | 9 | 6.29 | 44 | 24.44 |
| Memory (ability to remember things to do, some words, past and future events, etc.) | 4 | 2.80 | 17 | 9.44 |
| Upkeep of the house/flat | 4 | 2.80 | 7 | 3.89 |
| Atmosphere/ambience at school/university | 3 | 2.10 | 14 | 7.78 |
| Concentration and attention | 3 | 2.10 | 5 | 2.78 |
| Studies | 3 | 2.10 | 20 | 11.11 |
| Spiritual, philosophical or religious life | 2 | 1.40 | 5 | 2.78 |
| Your school/university results | 1 | 0.70 | 10 | 5.56 |
| Money available to you (pocket money, student job, allowance, etc.) | 1 | 0.70 | 16 | 8.89 |

| **Supplementary Table 5:** Life domains in which respondents consider themselves as "the unhappiest" | | | | |
| --- | --- | --- | --- | --- |
|  | **ALL survivors** | | **Populations controls** |  |
| **Domains ("the unhappiest")** | Number of respondent | % | Number of respondent | % |
| Peace of mind (not being worried, anxious or preoccupied or upset) | 39 | 27.27 | 50 | 27.78 |
| Self-esteem (overall opinion of yourself) | 34 | 23.78 | 56 | 31.11 |
| Financial Matters (incomes, allowance, etc.) | 33 | 23.08 | 43 | 23.89 |
| Sleep (ability to sleep well) | 33 | 23.08 | 49 | 27.22 |
| Concentration and attention | 28 | 19.58 | 40 | 22.22 |
| Upkeep of the house/flat | 24 | 16.78 | 31 | 17.22 |
| Love life/emotional life/life as a couple(signs of affection, understanding, communication) | 24 | 16.78 | 45 | 25.00 |
| Active leisure activities requiring energy (sport, gardening, etc.) | 23 | 16.08 | 26 | 14.44 |
| Vitality (Having energy to do things) | 23 | 16.08 | 37 | 20.56 |
| Moral | 22 | 15.38 | 25 | 13.89 |
| Memory (ability to remember things to do, some words, past and future events, etc.) | 21 | 14.69 | 17 | 9.44 |
| Physical abilities (ability to walk, climb stairs, etc.) | 21 | 14.69 | 29 | 16.11 |
| Absence of physical pain | 20 | 13.99 | 22 | 12.22 |
| Relaxing leisure activities (music, reading, cinema, going out, etc.) | 20 | 13.99 | 16 | 8.89 |
| Atmosphere/ambience at work | 19 | 13.29 | 29 | 16.11 |
| Intimate relations (libido, sexuality) | 19 | 13.29 | 35 | 19.44 |
| Overall physical health | 19 | 13.29 | 22 | 12.22 |
| Spiritual, philosophical or religious life | 17 | 11.89 | 19 | 10.56 |
| Interaction with your family (parents, siblings, etc.) | 14 | 9.79 | 15 | 8.33 |
| Paid work | 13 | 9.09 | 29 | 16.11 |
| Efficiency at work | 12 | 8.39 | 22 | 12.22 |
| Nutrition (type of food, etc.) | 10 | 6.99 | 25 | 13.89 |
| Interaction with your friends | 9 | 6.29 | 19 | 10.56 |
| Where you live (home, neighbourhood, surroundings, etc.) | 8 | 5.59 | 17 | 9.44 |
| Interaction with your children | 4 | 2.80 | 8 | 4.44 |
| Studies | 3 | 2.10 | 15 | 8.33 |
| Money available to you (pocket money, student job, allowance, etc.) | 3 | 2.10 | 42 | 23.33 |
| Your school/university results | 2 | 1.40 | 13 | 7.22 |
| Atmosphere/ambience at school/university | 1 | 0.70 | 13 | 7.22 |
